# Immunotherapy in older patients with non-small cell lung cancer: Young International Society of Geriatric Oncology position paper

**DOI:** 10.1038/s41416-020-0986-4

**Published:** 2020-07-22

**Authors:** Fabio Gomes, Melisa Wong, Nicolò Matteo Luca Battisti, Tiana Kordbacheh, Mandy Kiderlen, Alastair Greystoke, Andrea Luciani

**Affiliations:** 1 Medical Oncology, The Christie NHS Foundation Trust, Manchester, UK; 2 Division of Hematology/Oncology, University of California, San Francisco, CA USA; 3 Department of Medicine, The Royal Marsden NHS Foundation Trust, London, UK; 4 Division of Cancer Sciences, University of Manchester, Manchester, UK; 5 Radiation Oncology, Erasmus MC Cancer Institute, Rotterdam, Netherlands; 6 Medical Oncology, Newcastle-upon-Tyne NHS Foundation trust, Newcastle, UK; 7 Medical Oncology, Ospedale S. Paolo University Hospital, Milan, Italy

**Keywords:** Non-small-cell lung cancer, Lung cancer

## Abstract

Immunotherapy with checkpoint inhibitors against programmed cell death receptor (PD-1) and programmed cell death ligand (PD-L1) has been implemented in the treatment pathway of patients with non-small cell lung cancer (NSCLC) from locally advanced disease to the metastatic setting. This approach has resulted in improved survival and a more favourable toxicity profile when compared with chemotherapy. Following the successful introduction of single-agent immunotherapy, current clinical trials are focusing on combination treatments with chemotherapy or radiotherapy or even other immunotherapeutic agents. However, most of the data available from these trials are derived from, and therefore might be more applicable to younger and fitter patients rather than older and often frail lung cancer real-world patients. This article provides a detailed review of these immunotherapy agents with a focus on the data available regarding older NSCLC patients and makes recommendations to fill evidence gaps in this patient population.

## Background

More than half of all patients with non-small cell lung cancer (NSCLC) are aged above 70 years, and almost 10% are 80 years or older.^1^ The multi-organ age-related decline can alter drug pharmacokinetics and increase the risk of complications of locoregional and systemic treatments.^2,3^ This risk is also influenced by the increasing burden of comorbidities and polypharmacy, which increase the risk of adverse events and also impact survival.^4,5^ Moreover, quality of life (QoL) and functional endpoints are not well represented in clinical trials and should be considered at least as relevant as overall survival (OS).^6,7^

Chronological age alone provides relatively little information regarding the tolerance of older patients to cancer treatments. A comprehensive geriatric assessment (CGA), a multidisciplinary diagnostic and treatment process, can fill this knowledge gap and inform treatment decisions by identifying medical, psychosocial and functional limitations of older adults and facilitating a co-ordinated plan to maximise overall health in the context of ageing.^8^ In older cancer patients, the use of a CGA is associated with a number of benefits:^9,10^ the prediction of complications and side effects from treatment; estimation of survival; aiding patients, clinicians and family members in treatment decisions; detection of problems neglected by routine history and physical examination in the initial evaluation and new problems during follow-up care; improvement of mental health, well-being and pain control; and highlighting areas for potential intervention. Geriatric assessments have also been found to show prognostic value specifically in NSCLC patients.^11,12^ Furthermore, models based upon geriatric assessments have been developed to predict the risk of chemotherapy toxicity in older adults and better inform decision making.^13,14^ However, these assessments can be time-consuming and are not practical for all patients, and screening tools, such as G8, Flemish version of the Triage Risk Screening Tool and Vulnerable Elders Survey-13, have therefore been validated to identify those requiring a CGA.^15^

Appropriately selected older NSCLC patients have been shown to derive a similar survival benefit compared with their younger counterparts in the curative setting.^16,17^ Nonetheless, the underrepresentation of older adults in clinical trials defining the current standard of care limits the applicability of such results to the population seen in routine practice.^7,18^ In the palliative setting where chemotherapy is indicated, the decision-making should not be dictated by age alone.^19–21^ Single-agent chemotherapy can improve OS in older patients without adversely impacting QoL compared with best supportive care alone;^22–24^ data are controversial regarding the benefit of combination chemotherapy in this age group, particularly in those who are more frail.^21,25^ Tyrosine kinase inhibitors (TKIs) such as those targeting the epidermal growth factor receptor (EGFR), anaplastic lymphoma kinase (ALK) or ROS-1 are the treatment of choice for oncogene-addicted NSCLC patients, on the basis of the superiority of these agents in survival outcomes and their mild toxicity profile. Although TKIs are often a good match for older patients, these patients constitute a small subset of NSCLC and might still be at a higher risk of toxicity.

Immune checkpoint inhibitors, designed to revitalise antitumour immune responses, have revolutionised the management of a number of malignancies, including NSCLC; this type of immunotherapy also represents a potentially appropriate treatment option for older patients. Below, we outline the mechanism of action of immunotherapy and its adverse events before reviewing the data supporting the use of immunotherapy in patients with NSCLC—alone or in combination—with a particular focus on older patients, in an effort to address the issue of whether age influences the efficacy and toxicity of this approach. We also discuss the potential impact of the ageing process on the immune system and, hence, on the efficacy of immunotherapy.

## Immunotherapy

### Mechanism of action of immune checkpoint inhibitors

Strict regulation of the immune system is crucial for allowing the co-ordinated clearance of infected or malignant cells while sparing normal cells. In addition, mechanisms to downregulate the immune response are important to prevent immune over-reactivity once a pathogenic insult has been cleared and in cases where cells different from self are encountered in a physiological setting, such as in gamete formation or in the developing foetus.^26^

Evading immunosurveillance is one of the hallmarks of cancer—cancer cells hijack the key regulatory mechanisms of the immune system, such as checkpoint pathways, to enable their survival.^27^ Immune checkpoint pathways operate during homoeostasis to control the duration and extent of immune responses and prevent autoimmunity, but tumour cells have developed the ability to activate inhibitory checkpoints on T cells to avoid being recognised and destroyed. The importance of inhibitory checkpoint signals on T cells in immune evasion led to the development of two classes of inhibitory monoclonal antibody, which are now standard treatment options for a number of malignancies including NSCLC: those that block the interaction between cytotoxic T-lymphocyte associated protein 4 (CTLA4) on the tumour and B7 on the T cell that inhibits T-cell priming activation; and those that block the interaction between programmed death receptor-ligand 1 (PD-L1) on the tumour and programmed death receptor 1 (PD-1) on the T cell that inhibits recognition of the tumour cells by T cell and subsequent tumour cell lysis.^28^ Ongoing research is investigating the role of multiple targets in thoracic malignancies, including other stimulatory/inhibitory receptors involved in T-cell checkpoints and the use of novel agents in combination with currently licensed agents.^26,29^

### Treatment-related adverse events

Immunotherapy is associated with a unique spectrum of treatment-related adverse events (TRAEs), also known as immune-related adverse events. These include dermatological, gastrointestinal, hepatic, endocrine and other less common inflammatory events arising from general immunologic enhancement.^30^ Older patients often have an increased risk of TRAEs with cancer treatments in general due to a decreased organ reserve, comorbidities and polypharmacy. In the case of immunotherapy, an aged immune system may, in principle, play an additional important role in determining the risk of TRAEs.

## Immunosenescence

Older age correlates with a decline in organ function,^31^ including the composition and function of the immune system—its cells, the microenvironment in which they operate and the cytokines modulating their proliferation and activity.^32^ This decline might, in principle, result in an altered efficacy and safety profile of immunotherapy agents in the older cancer patient.

The remodelling of the immune system associated with the ageing process is called immunosenescence^32^ and involves a number of changes that can be associated with a decrease in immune surveillance both in the adaptive and innate immune system. In older patients, this reduced surveillance manifests clinically as an increased risk of developing viral and bacterial infections and reactivation of latent infections, such as varicella zoster virus and cytomegalovirus (CMV).^33,34^ Chemotaxis, phagocytosis and cytotoxicity are impaired, as are the mechanisms of antigen presentation by macrophages and dendritic cells.^35^ The responsiveness of T cells to pathogens decreases with age and involves a reduced ability to move to lymph nodes, lower proliferation in response to antigens and cytokines and reduced cytokine release. These changes result in the loss of the co-stimulatory protein CD28, particularly in CD8 lymphocytes.^36^ CD8^+^CD28^–^ lymphocytes downregulate responses (suppressor effect) via CD4^+^ cells and dendritic cells, and are often clonally expanded, thereby reducing the numbers of both naïve and central memory T cells. The impact of recurrent infections—in particular, CMV infections—on naïve T cells is deemed to be a key contributor to these changes.^37^ Interestingly, CD8^+^CD28^–^ lymphocytes gain other functions, showing increased cytotoxicity mediated by enzymes usually found in natural killer cells.^38^

Immunotherapy toxicity may occur as a process of autoimmunity. Although higher levels of autoantibodies are seen in older patients, it is still unclear whether this change translates into an increased risk of side effects from immunotherapy agents.^39^ Additionally, it has been suggested that older adults also have higher levels of myeloid-derived suppressor cells and regulatory T (T_reg_) cells,^40,41^ which are key mediators of immune evasion and resistance to checkpoint inhibitors. Older age is associated with higher levels of systemic inflammation, with increased levels of pro-inflammatory cytokines such as interleukin (IL)-6 and acute-phase proteins such as C-reactive protein (CRP), a phenomenon often called ‘inflammaging’.^42^ While high levels of IL-6 in the tumour microenvironment are associated with resistance to checkpoint inhibitors,^26,43^ more research is needed on the implications of inflammaging on outcomes of immunotherapy.^32^ Finally, age also influences the interaction between the microbiome and immune system. Animal models and clinical series suggest that changes in the microbiome influence the efficacy of checkpoint inhibition;^44^ consequently, the decline in microbiota diversity associated with ageing might negatively influence immune checkpoint inhibitors.^45^

## Single-agent immunotherapy

As immunotherapy with immune checkpoint inhibitors started revolutionising the treatment of NSCLC, the first step was the development of monotherapy agents.

### Pembrolizumab

This anti-PD-1 monoclonal antibody was the first checkpoint inhibitor agent to be investigated for the management of patients with advanced NSCLC. The Phase 3, randomised KEYNOTE-010 trial investigated the use of pembrolizumab versus docetaxel in pretreated patients with PD-L1 expression on at least 1% of tumour cells.^46^ The median OS was 10.4 versus 8.5 months, favouring pembrolizumab (hazard ratio [HR] 0.71, 95% confidence interval [CI] 0.58–0.88; *P* = 0.0008), and higher levels of PD-L1 expression on tumour cells were associated with better outcomes (HR 0.54, 95% CI 0.38–0.77; *P* = 0.0002 in the PD-L1 > 50% subgroup). In this setting, the median OS improvement was 13% inferior for patients aged ≥65 years (Table 1) but there was only a small proportion of patients in that upper age cohort, which limits any conclusions.

**Table 1:** Summary of data from immunotherapy single-agent trials for NSCLC.

Study	Design and setting	Trial arms	*N* and age (years)	Key findings in older adults
*Pembrolizumab*				
KEYNOTE-024 NCT02142738	Phase 3 First line Squamous and non-squamous PD-L1 > 50%	Pembrolizumab versus platinum-based chemotherapy	*n* = 305 (1:1) Median age 65 (range 33–90) Age ≥65: 54%	OS ≥ 65y: HR 0.64 (95% CI 0.42–0.98) OS < 65y: HR 0.60 (95% CI 0.38–0.96)
KEYNOTE-042 NCT02220894	Phase 3 First line Squamous and non-squamous PD-L1 > 1%	Pembrolizumab versus platinum-based chemotherapy	*n* = 1274 (1:1) Median age 63 (range 25–90) Age ≥65: 45%	OS ≥ 65y: HR 0.82 (95% CI 0.66–1.01) OS < 65y: HR 0.81 (95% CI 0.67–0.98)
KEYNOTE-010 NCT01905657	Phase 3 Second line and subsequent Squamous and non-squamous PD-L1 > 1%	Pembrolizumab versus Docetaxel	*n* = 1034 (1:2) Median age 63 (range 54–70) Age ≥65: 41%	OS ≥ 65y: HR 0.76 (95% CI 0.57–1.02) OS < 65y: HR 0.63 (95% CI 0.50–0.79)
*Nivolumab*				
CHECKMATE-026 NCT02041533	Phase 3 First line Squamous and non-squamous PD-L1 > 1%	Nivolumab versus platinum-based chemotherapy	*n* = 423 (1:1) Median age 64 (range 29–89) Age ≥65: 48%	OS ≥ 65y: HR 1.04 (95% CI 0.77–1.41) OS < 65y: HR 1.13 (95% CI 0.83–1.54)
CHECKMATE-017 NCT01642004	Phase 3 Second line and subsequent Squamous	Nivolumab versus Docetaxel	*n* = 272 (1:1) Median age 63 (range 39–85) Age ≥65: 44%	OS ≥ 75y: HR 1.85 (95% CI 0.76–4.51) OS 65–74: HR 0.56 (95% CI 0.34–0.91) OS < 65y: HR 0.52 (95% CI 0.35–0.75)
CHECKMATE-057 NCT01673867	Phase 3 Second line and subsequent Non-squamous	Nivolumab versus Docetaxel	*n* = 582 (1:1) Median age 62 (range 21–85) Age ≥65: 42%	OS ≥ 75y: HR 0.90 (95% CI 0.43–1.87) OS 65–74: HR 0.63 (95% CI 0.45–0.89) OS < 65y: HR 0.81 (95% CI 0.62–1.04)
*Atezolizumab*				
OAK NCT02008227	Phase 3 Second line and subsequent Squamous and non-squamous	Atezolizumab versus Docetaxel	*n* = 850 (1:1) Median age 64 (range 33–85) Age ≥65: 47%	OS ≥ 65y: HR 0.66 (95% CI 0.52–0.83) OS < 65y: HR 0.80 (95% CI 0.64–1.00)
*Durvalumab*				
MYSTIC NCT02453282	Phase 3 First line Squamous and non-squamous	Durvalumab versus platinum-based chemotherapy (versus Durvalumab/ tremelimumab)	*n* = 1118 (1:1:1) Median age 65 (range 32–87) Age ≥65: NR	Durvalumab versus chemotherapy OS ≥ 65y: HR 0.66 (95% CI 0.45–0.95) OS < 65y: HR 0.86 (95% CI 0.60–1.24)
PACIFIC NCT02125461	Phase 3 Stage III Adjuvant post CCRT Squamous and non-squamous	Durvalumab versus placebo	*n* = 709 (1:2) Median age 64 (range 23–90) Age ≥65: 45%	OS ≥ 65y: HR 0.76 (95% CI 0.55–1.06) OS < 65y: HR 0.62 (95% CI 0.44–0.86)

*CCRT* concurrent chemoradiotherapy, *CI* confidence interval, *HR* hazard ratio, *NR* not reported, *OS* overall survival, *PD-L1* programmed cell death ligand 1, *y* years.

In the first-line setting, the Phase 3 KEYNOTE-024 trial randomised NSCLC patients with tumour PD-L1 expression of >50% to pembrolizumab versus standard-of-care platinum-based chemotherapy.^45^ The median OS was 30 versus 14.2 months, favouring pembrolizumab (death HR 0.49, 95% CI 0.34–0.69, adjusted for crossover). The OS benefit was consistent across subgroups (Table 1). The 3-year survival update confirmed the durable survival benefit of pembrolizumab, with 43.7% of patients alive versus 24.9% on the chemotherapy arm (death HR 0.65, 95% CI 0.50–0.86; *P* < 0.01).^46^

The Phase 3 randomised KEYNOTE-042 trial had a similar design and treatment arms but randomised patients with tumour PD-L1 expression >1%.^47^ The median OS was superior for the pembrolizumab arm at different PD-L1 expression cut-offs (>1, >20 and >50%), although the magnitude of benefit was smaller in the case of lower PD-L1 expression (HR 0.81, 95% CI 0.71–0.93, *P* = 0.0018, for >1% expression versus HR 0.69, 95% CI 0.56–0.85, *P* = 0.0003 for >50% expression). Moreover, there was no benefit with pembrolizumab when explored in the subgroup of PD-L1 1–49%. With regard to older patients, the OS benefit was similar across subgroups (Table 1).

No age-specific data on toxicity are available from these three trials but the overall incidence of TRAEs of grades 3–5 varied between 13 and 31% with pembrolizumab versus 35 and 53% with chemotherapy.^45,47,48^ A 2019 pooled analysis of the above-mentioned Phase 3 trials focused on the efficacy and safety in patients aged 75 years or above and confirmed an OS benefit of pembrolizumab (tumour PD-L1 expression of either ≥1 or ≥50%) versus chemotherapy, with a favourable toxicity profile, similar to their younger counterparts.^49,50^

### Nivolumab

The anti-PD-1 monoclonal antibody nivolumab was first evaluated in two Phase 3 trials in patients who had previously been treated with platinum doublet chemotherapy. The CHECKMATE-017 and CHECKMATE-057 trials randomised patients regardless of PD-L1 expression to nivolumab versus docetaxel for squamous and non-squamous NSCLC subtypes, respectively.^51,52^ Several pooled analyses of both trials with increasing follow-up periods have been published: the 5-year pooled analysis represents the longest survival follow-up with immunotherapy for randomised Phase 3 trials in patients with advanced NSCLC.^53–56^ This latest analysis confirmed the long-term OS benefit of nivolumab (HR 0.68, 95% CI 0.59–0.78) with an OS rate at 5 years of 13% versus 3% with docetaxel.^55^ In the subgroup analysis, the benefit of nivolumab for patients aged 75 years or above was not clearly established considering the small number of patients within this age group in both trials (Table 1). The use of nivolumab as monotherapy had an incidence of TRAEs of grade 3–5 of 10% in the nivolumab pooled analysis compared with 55% for docetaxel.

In the CHECKMATE-026 trial, nivolumab was compared with the standard of care first-line platinum-based chemotherapy for patients with PD-L1 expression ≥1%.^57^ This trial was negative regarding progression-free survival (PFS), which was its primary endpoint. The Phase 2 CHECKMATE-171 trial evaluated the safety of nivolumab in a European population of pretreated patients with squamous NSCLC^58^ and reported an incidence of grade 3–4 TRAEs for those aged ≥70 years of 14%, compared with 12% across the study population. Similarly, the Phase 3b/4 CHECKMATE-153 trial assessed the safety profile of nivolumab in North America and reported an incidence of grade 3–4 TRAEs of 12% for those aged ≥70 years compared with 11% for younger patients.^59^

### Atezolizumab

This anti-PD-L1 monoclonal antibody was explored as monotherapy versus docetaxel in the Phase 3 OAK trial in pretreated NSCLC patients regardless of their PD-L1 expression.^60^ The median OS was 13.8 months on atezolizumab compared with 9.6 months on docetaxel (HR 0.73, 95% CI 0.62–0.87; *P* = 0.0003). In the subgroup analysis, older patients (≥65 years) had an additional 14% reduction in the risk of death compared with younger patients (Table 1). No age-specific safety data are available, although the incidence of grade 3–5 TRAEs was 15% for atezolizumab versus 43% with docetaxel. Moreover, the use of atezolizumab delayed the time to deterioration in physical function in the study population (HR 0.75, 95% CI 0.58–0.98).^61^ Considering that the lung cancer population is predominantly older, with 44% of cases in the UK occurring in patients aged 75 and older, a benefit on physical function is of great clinical significance.^62^ Data on the use of single-agent atezolizumab in the first-line setting from IMPOWER-110 (NCT02409342) and IMPOWER-111 (NCT02409355) trials are awaited.

### Durvalumab

The Phase 3 MYSTIC trial investigated durvalumab versus platinum-based chemotherapy versus the combination of durvalumab and tremelimumab, a monoclonal anti-CTLA-4 antibody, in the first-line setting.^63^ In the subgroup of patients with PD-L1 expression ≥25% (primary analysis subgroup), the median OS for durvalumab versus chemotherapy was 16.3 versus 12.9 months, respectively (HR 0.76, 97.5% CI 0.56–1.02; *P* = 0.036)—although statistical significance was not achieved, this constitutes a clinically meaningful improvement in OS for durvalumab versus chemotherapy. A more meaningful benefit for older patients (65 years or older) was observed, with HR 0.66 (97.5% CI 0.45–0.95) favoring durvalumab over chemotherapy.^64^ When comparing durvalumab plus tremelimumab with chemotherapy, the median OS was 11.9 versus 12.9 months (HR 0.85, 98.8% CI 0.61–1.17; *P* = 0.202) with no benefit in any age groups.^63,64^ With regard to safety, the incidence of TRAEs of grades 3–5 was 15% with durvalumab versus 35% with chemotherapy. No age-group analyses of TRAEs were carried out.

## Chemoimmunotherapy

Chemotherapy coadministered with immunotherapy is a more recent development in the management of patients with advanced NSCLC. A number of reasons exist for potentially better outcomes on a combination. Cytotoxic cell death might create additional antigens that are recognised by the immune system.^65^ In addition, chemotherapeutic agents can reduce the number of suppressive cells, such as myeloid-derived suppressor cells and T_reg_ cells, that would otherwise limit the efficacy of the immunotherapeutic agents.^66^ Furthermore, by reducing the tumour bulk, cytotoxic agents allow T-lymphocytes to infiltrate the tumour and recovery of an exhausted immune.^67^

### Pembrolizumab combinations

In KEYNOTE-189,^68^ a Phase 3 double-blind, randomised placebo-controlled trial of patients with metastatic non-squamous NSCLC and any level of tumour PD-L1 expression, first-line pembrolizumab plus platinum-based chemotherapy (cisplatin or carboplatin) and pemetrexed was superior to platinum-based chemotherapy and pemetrexed in terms of OS (overall HR 0.49, 95% CI 0.38–0.64) and PFS (overall HR 0.52, 95% CI 0.43–0.64). Median OS in the chemoimmunotherapy arm was 22.0 months, versus 10.7 months for the standard chemotherapy arm (HR 0.56, 95% CI 0.45–0.70; *P* < 0.01).^69^ In subgroup analyses by age (Table 2), the OS benefit extended to patients of 65 years and over (HR 0.64, 95% CI 0.43–0.95).^68^ No subgroup analyses by age were conducted for PFS or for any toxicity outcomes. In the chemoimmunotherapy arm, 67.2% of patients of all ages developed TRAEs of grade 3 and above, compared with 65.8% in the chemotherapy arm. The results from KEYNOTE-189^68^ and its predecessor Phase 2 KEYNOTE-021 trial^70,71^ led to the widespread approval of pembrolizumab in combination with platinum and pemetrexed for the first-line treatment of metastatic non-squamous NSCLC in patients without EGFR or ALK genomic tumour aberrations, regardless of PD-L1 expression.

**Table 2:** Summary of data from combination trials of systemic treatments for NSCLC.

Study	Design and setting	Trial arms	*N* and age (years)	Key findings in older adults
*Pembrolizumab plus chemotherapy*				
KEYNOTE-189 NCT02578680	Phase 3 First line non-squamous PD-L1 (any)	Cisplatin or carboplatin, pemetrexed (maintenance pemetrexed) versus Cisplatin or carboplatin, pemetrexed + pembrolizumab (maintenance pemetrexed + pembrolizumab)	*n* = 616 (1:2) Median age 64 (range 34–84) Age ≥65: 49%	OS > 65y: HR 0.64 (95% CI 0.43–0.95) OS < 65y: HR 0.43 (95% CI 0.31–0.61)
KEYNOTE-407 NCT02775435	Phase 3 First line squamous PD-L1 (any)	Carboplatin, (nab)-paclitaxel (maintenance pemetrexed) versus Carboplatin, (nab)-paclitaxel + pembrolizumab (maintenance pemetrexed + pembrolizumab)	*n* = 559 (1:1) Median age 65 (range 29–88) Age ≥65: 55%	OS > 65y: HR 0.74 (95% CI 0.51–1.07) OS < 65y: HR 0.52 (95% CI 0.34–0.80) PFS > 65y: HR 0.63 (95% CI 0.47–0.84) PFS < 65y: HR 0.50 (95% CI 0.37–0.69)
*Atezolizumab plus chemotherapy*				
IMpower150 NCT02366143	Phase 3 First line EGFR/ALK^+^ allowed after >1 TKI non-squamous PD-L1 (any)	Carboplatin, paclitaxel, bevacizumab (maintenance bevacizumab) versus Carboplatin, paclitaxel, bevacizumab + atezolizumab (maintenance bevacizumab + atezolizumab) (versus Carboplatin, paclitaxel, atezolizumab; results from this arm not reported)	*n* = 800 (1:1) Median age 63 (range 31–90) Age ≥65: 45%	PFS ≥ 75y: HR 0.78 (95% CI NR) PFS 65–74y: HR 0.52 (95% CI NR) PFS < 65y: HR 0.65 (95% CI NR)
IMpower130 NCT02367781	Phase 3 First line EGFR/ALK^+^ allowed after >1 TKI Non-squamous PD-L1 (any)	Carboplatin, nab-paclitaxel, (best supportive care or switch maintenance pemetrexed) versus Carboplatin, nab-paclitaxel + atezolizumab (maintenance atezolizumab)	*n* = 723 (1:2) Age ≥65: 50%	OS ≥ 65y: HR 0.78 (95% CI 0.58–1.05) OS < 65y: HR 0.79 (95% CI 0.58–1.08) PFS ≥ 65y: HR 0.64 (95% CI 0.50–0.82) PFS < 65y: HR 0.64 (95% CI 0.50–0.82)
IMpower132 NCT02657434	Phase 3 First line Non-squamous PD-L1 (any)	Carboplatin or cisplatin, pemetrexed (maintenance pemetrexed) versus Carboplatin or cisplatin, pemetrexed + atezolizumab (maintenance pemetrexed + atezolizumab)	*n* = 578 (1:1) Age ≥65: 45%	OS ≥ 65y: HR 0.71 (95% CI 0.50–1.01) OS < 65y: HR 0.89 (95% CI 0.65–1.21) PFS ≥ 65y: HR 0.55 (95% CI 0.42–0.73) PFS < 65y: HR 0.63 (95% CI 0.49–0.80)
IMpower131 NCT02367794	Phase 3 First line squamous PD-L1 (any)	Carboplatin, nab-paclitaxel versus Carboplatin, nab-paclitaxel + atezolizumab (maintenance atezolizumab) (versus Carboplatin, paclitaxel + atezolizumab (maintenance atezolizumab); results from this arm not reported)	*n* = 1021 (1:1) Median age 65 (range 23–86) Age ≥65: 52%	PFS ≥ 75y: HR 0.51 (95% CI 0.30–0.84) PFS 65–74y: HR 0.66 (95% CI 0.51–0.87) PFS < 65y: HR 0.77 (95% CI 0.61–0.99)
*Nivolumab plus Ipilimumab*				
CheckMate-227 NCT02477826 (Part 1—group PD-L1≥1% only)	Phase 3 First line squamous and non-squamous PD-L1 ≥1%	Nivolumab + Ipilimumab versus platinum-based chemotherapy (versus Nivolumab—not included in primary endpoint analysis)	*n* = 1189 (1:1:1) Median age 64 (range 26–87) Age ≥65: 49%	OS ≥ 75y: HR 0.92 (95% CI 0.57–1.48) OS 65–74y: HR 0.91 (95% CI 0.70–1.19) OS < 65y: HR 0.70 (95% CI 0.55–0.89)
CheckMate-817 NCT02869789 (Cohorts A and A1)	Phase 3b/4 First line squamous and non-squamous PD-L1 (any)	Nivolumab + Ipilimumab	*n* = 391 + 198 Median age 65 (range 26–90) Age ≥65: NR	NR
Lung-MAP NCT02785952 Sub-study S1400I	Phase 3 Second line squamous PD-L1 (any)	Nivolumab + Ipilimumab versus Nivolumab	*n* = 275 (1:1) Median age and range NR Age ≥65: NR	NR
*Durvalumab plus Tremelimumab*				
MYSTIC NCT02453282	Phase 3 First line squamous and non-squamous	(Durvalumab versus) Durvalumab/tremelimumab versus platinum-based chemotherapy	*n* = 1118 (1:1:1) Median age 65 (range 32–87) Age ≥65: NR	Durvalumab/tremelimumab versus chemotherapy OS ≥ 65y: HR 0.72 (95% CI 0.50–1.02) OS < 65y: HR 1.01 (95% CI 0.70–1.46)

*CI* confidence interval, *IQR* interquartile range, *HR* hazard ratio, *NR* not reported, *OS* overall survival, *PD-L1* programmed cell death ligand 1, *PFS* progression-free survival, *TKI* tyrosine kinase inhibitor, *y* years.

For patients with metastatic squamous NSCLC, only one landmark trial to date has demonstrated an OS benefit for chemoimmunotherapy compared with chemotherapy. In KEYNOTE-407,^72,73^ a Phase 3 double-blind randomised placebo-controlled trial of patients with metastatic squamous NSCLC and any level of tumour cell PD-L1 expression, first-line treatment with pembrolizumab plus carboplatin plus either paclitaxel or nab-paclitaxel was superior to carboplatin plus taxane alone in terms of OS (17.1 versus 11.6 months; HR 0.71, 95% CI 0.58–0.88) and PFS (8.0 versus 5.1 months; HR 0.57, 95% CI 0.47–0.69). In subgroup analyses by age, the PFS benefit extended to older adults (age > 65 years, HR 0.63, 95% CI 0.47–0.84). However, when OS was examined among older adults only, there was no longer a statistically significant benefit (age > 65 years, HR 0.74, 95% CI 0.51–1.07). Overall, TRAEs of grade 3 and above occurred similarly in both arms, affecting 69.8% of patients receiving chemoimmunotherapy and 68.2% of patients receiving chemotherapy. No age-specific data on toxicity are available.

### Atezolizumab combinations

In IMpower150,^74^ an open-label Phase 3 randomised trial of patients with metastatic non-squamous NSCLC and any level of tumour cell PD-L1 expression (including patients with EGFR or ALK genetic alterations), a combination of carboplatin, paclitaxel, bevacizumab plus atezolizumab was superior to carboplatin, paclitaxel and bevacizumab with respect to OS (overall HR 0.78, 95% CI 0.64–0.96) and PFS (overall HR 0.62, 95% CI 0.52–0.74). In a comparison of age groups, PFS was favoured by the chemoimmunotherapy arm in patients below the age of 65 years (HR 0.65) and in those aged 65–74 years (HR 0.52). Among patients aged 75–84 (9% of patients), the HR for PFS was 0.78 but was not statistically significant. The results comparing an additional third arm consisting of carboplatin and paclitaxel plus atezolizumab have not yet been reported. Overall, grade >3 TRAEs were reported in 58.5% of patients in the chemoimmunotherapy arm and 50% in the chemotherapy arm.

In the IMpower130 trial, atezolizumab was also studied in combination with carboplatin and nab-paclitaxel compared with chemotherapy alone in patients with metastatic non-squamous NSCLC,^75^. PFS (HR 0.65, 95% CI 0.54–0.77) and OS (HR 0.80, 95% CI 0.65–0.99) were improved in the chemoimmunotherapy arm in the intention-to-treat population. When analysed by age group, the PFS benefit of the chemoimmunotherapy arm remained and was similar among younger and older patients (age < 65 years PFS HR 0.64, 95% CI 0.50–0.82; age >65 years PFS HR 0.64, 95% CI 0.50–0.82). By contrast, the OS benefit of the chemoimmunotherapy arm was no longer statistically significant when stratified by age group (age < 65 years OS HR 0.79, 95% CI 0.58–1.08; age >65 years OS HR 0.78, 95% CI 0.58–1.05). Grade >3 TRAEs occurred in 75% of patients in the chemoimmunotherapy arm and 60% in the chemotherapy arm.

Atezolizumab was also studied in patients with metastatic non-squamous NSCLC in combination with carboplatin or cisplatin plus pemetrexed versus chemotherapy alone in the IMpower132 trial.^76^ PFS (HR 0.60, 95% CI 0.49–0.72) was improved in the chemoimmunotherapy arm compared with the chemotherapy arm, and this improvement was confirmed in age-group analyses as well. Among older patients aged >65 years, the HR for PFS was 0.55 (95% CI 0.42–0.73) compared with a HR of 0.63 (95% CI 0.49–0.80) for younger patients. In the oldest group (aged 75–84 years), the PFS HR was 0.63 (95% CI 0.35–1.13). The interim OS analysis did not demonstrate a benefit at this time (HR 0.81, 95% CI 0.64–1.03), which was not influenced by age group (age < 65 OS HR 0.89, 95% CI 0.65–1.21; age >65 OS HR 0.71, 95% CI 0.50–1.01).

For patients with metastatic squamous NSCLC, the open-label Phase 3 randomised trial IMpower131^77^ demonstrated a PFS benefit for first-line atezolizumab plus carboplatin and nab-paclitaxel versus carboplatin and nab-paclitaxel alone (HR 0.71, 95% CI 0.60–0.85). The final OS data showed no benefit for the intent-to-treat population (HR 0.88, 95% CI 0.73–1.05; *P* = 0.158), but on secondary analysis for those with high PD-L1 expression (≥50% PD-L1 expression on tumour cells or ≥10% expression on tumour-infiltrating immune cells) there was an apparent benefit in OS (HR 0.48, 95% CI 0.29–0.81).^78^ In subgroup analyses by age, only available for PFS, a benefit to all three age groups was demonstrated (age < 65 years HR 0.77, 95% CI 0.61–0.99; age 65–74 years HR 0.66, 95% CI 0.51–0.87; age 75–84 years HR 0.51, 95% CI 0.30–0.84). TRAEs of grade 3 and above occurred in 69% of patients in the chemoimmunotherapy arm compared with 58% in the chemotherapy arm.

## Combinations of immunotherapy

Combining different immunotherapy agents that target different checkpoints in T cells is the most recent development in the field of advanced NSCLC. CHECKMATE-227 is a complex randomised Phase 3 trial divided into two parts for the first-line treatment of patients with advanced NSCLC primarily exploring the combination of nivolumab plus ipilimumab versus standard platinum-based chemotherapy. The first part, which has been published, had two independent primary endpoints: PFS with nivolumab plus ipilimumab versus chemotherapy in patients with a high tumour mutational burden (≥10 mutations per megabase);^79^ and OS with nivolumab plus ipilimumab versus chemotherapy in patients with a tumour PD-L1 expression level of 1% or more.^80^ For other hierarchical endpoints, the trial included a group with PD-L1 expression below 1% and also treatment arms with nivolumab or nivolumab plus chemotherapy. Focusing on the published data for the primary OS endpoint in the case of PD-L1 expression levels ≥1%, the nivolumab plus ipilimumab combination was superior to the chemotherapy arm (17.1 versus 14.9 months; HR 0.79, 95% CI 0.65–0.96; *P* = 0.007). In the subgroup analysis, the benefit for the group aged 65–74 years was not clear when compared with younger patients, with a HR of 0.91 (0.70–1.19) versus HR 0.70 (0.55–0.89), respectively. Similarly, the group aged 75 years or more did not seem to benefit, although this was a small group comprising only 81 patients. With regard to toxicity, grade >3 TRAEs were reported in 32.8% of patients in the nivolumab plus ipilimumab arm and 36% in the chemotherapy arm, with more serious adverse events occurring in the immunotherapy arm (24.5% versus 13.9%).

CHECKMATE-817 is a Phase 3b/4 trial primarily exploring the safety (grade 3–5 TRAEs) of a flat dose of nivolumab combined with ipilimumab (standard weight-based dose) in the first-line treatment of advanced NSCLC. The trial included two cohorts: a standard cohort of 391 patients with a performance status (PS) of 0–1, and a smaller, ‘special populations’ cohort of 198 patients comprising those with a PS of 2 or of 0–1 plus other factors that might have excluded them from other clinical studies of immunotherapy agents (asymptomatic untreated brain metastasis, hepatic impairment, renal impairment or human immunodeficiency virus).^81,82^ In the main cohort (those with PS 0–1), 15% was aged 75 years or above, whilst 22% of the PS 2 group within the special populations’ cohort (comprising 139 patients) was aged 75 years or above. The incidence of grade 3–4 TRAEs was 35% and 26%, respectively, favouring the older, more frail group, with no difference in treatment-related death. Moreover, the overall safety data of nivolumab flat-dosing was identical to the weight-based dosing modality.

## Radioimmunotherapy

Between 30 and 50% of patients diagnosed with NSCLC receive radiotherapy in the early- or late-stage disease setting and, as such, radiotherapy is a valuable treatment modality. Radiotherapy is known to induce immune and inflammatory changes that can prime the tumour microenvironment to initiate an immune response. Moreover, this initial immune priming can be augmented systemically by combining radiotherapy with immunotherapies to relay an abscopal response. Radiation-induced immunogenic cell death induces the release of tumour antigens, dendritic cell maturation, augmentation of T-cell priming, upregulation of MHC-I and PD-L1 expression, and upregulation of the levels of cytokines and chemokines^83–87^—consequently, interest in combining radiotherapy with immunotherapy agents to improve antitumour immunity and responses has increased.

Clinical evidence on the combination of thoracic radiotherapy and immunotherapy for patients with NSCLC is lacking. However, some data are available on their sequential use.^88^ A secondary analysis of KEYNOTE-001, a Phase 1 study that included patients with metastatic NSCLC, showed that those who previously received radiotherapy had a significantly longer PFS and OS than non-irradiated patients when subsequently treated with pembrolizumab.^88^ However, these patients also experienced a greater degree of pulmonary toxicity compared with non-irradiated patients. Although increasing age was associated with improved PFS in this model on univariate analysis; it no longer reached significance on multivariate analysis, which may be linked with the presence of clinical confounding factors.

The efficacy of durvalumab in the setting of unresectable stage III NSCLC following concurrent chemoradiotherapy was explored in the PACIFIC trial.^89^ Durvalumab significantly prolonged the median OS compared with placebo (HR 0.68, 99.7% CI, 0.47–0.997; *P* = 0.0025). Nevertheless, the OS benefit was less clear for older patients (Table 2). In this trial, the incidence of pneumonitis of any grade was higher in the durvalumab arm than in the placebo (34% versus 25%), although the rates of grade 3–4 pneumonitis were similar (4% versus 3%). An exploratory analysis investigated the efficacy of durvalumab in patients who developed pneumonitis, and the survival outcomes were similar to the intent-to-treat population.^90^ Although radiation pneumonitis becomes more common with age,^91^ this does not appear to be the case for immunotherapy pneumonitis.^92^

## Discussion

Immune checkpoint inhibition therapy targeting PD-1/PD-L1 has changed the treatment landscape for advanced NSCLC. Although due to the ageing immune system (immunosenescence) there is a concern that older patients might be at risk of lower efficacy and/or increased adverse effects with these agents, limited subgroup analyses from pivotal clinical trials indicate that older patients might gain the same benefit from immunotherapy as younger patients, with an acceptable toxicity profile.

However, methodologically and conceptually, results from the pivotal Phase 3 trials cannot yet be generalised to older patient populations. These trials only included patients with a PS of 0–1; consequently, the evidence in vulnerable/frail patients remains very limited. The median age at trial enrolment was about 10 years younger than the median age of NSCLC diagnosis in Western countries. The subgroup analyses on older patients were conducted post-hoc and the trials not powered for age-group comparison. Data on patients >75 years old are lacking, and any available data in this group are conflicting, potentially reflecting the small sample size of these elderly cohorts, or poorer tolerance of therapy and additional comorbidities within this population.

Moreover, the pivotal Phase 3 trials did not include CGA or geriatric screening at baseline as suggested by current guidelines.^93^ Prospective studies with a real-world population are therefore required to incorporate geriatric assessments into their design, such as is the case in the ELDERS study. This observational cohort study of 140 patients (with NSCLC or melanoma) was designed with the primary aim of assessing safety, but also to investigate the QoL of immunotherapy in younger and older patients (cut-off at 70 years). The PS value was not part of the selection criteria and the study integrated geriatric screening and assessments for subsequent exploratory analysis. An interim analysis of the first 32 patients with NSCLC reported no significant differences in toxicity between the age groups in a real-world population where 30% of patients were PS 2 and 46% of the older patients failed the geriatric screening (using the G8 tool).^94^ The final results of this study are expected in late 2020.

It is still not entirely clear whether the poorer PS and increased incidence of comorbidities that can be associated with older age predict for more toxicity and/or less efficacy with immunotherapy. In two large cohort studies of nivolumab, patients with PS 2 had similar adverse events compared with PS 0–1 but poorer outcomes in terms of OS.^95,96^ However, these results were not reproduced in the PePS2 study assessing pembrolizumab in a PS 2 population, in which the response and OS appeared similar to previous reports in patients with PS 0–1.^97^ Additionally, the CheckMate-171 trial, which included elderly and PS 2 patients, reported no differences in terms of toxicity and OS between the overall population and the elderly subgroup.^58^ Real-world data derived from the Italian expanded access programme (EAP) for nivolumab in pretreated patients also suggested a similar OS across age groups and, although toxicities were not analysed separately, their overall incidence was similar to data derived from randomised trials.^98–100^ Further data from an Italian multicentre retrospective study of patients >75 years old treated with anti-PD-1 agents (either nivolumab or pembrolizumab) were also consistent with previous registration trials in terms of toxicity profile and efficacy.^101^ Therefore, there is currently no need to exclude patients with reasonable performance status from treatment with immunotherapy on the basis of age.

Contrary to chemotherapy, the duration of treatment with immunotherapy is long, and patients can receive treatment for many months or even years. However, the impact of long-term treatment on patient fitness and comorbidities is unclear. In patients experiencing immune-related adverse effects, steroids are recommended—often at high doses and for prolonged courses.^102^ The impact on older patients of managing these effects is not clear but might be as problematical as immunotherapy treatment itself, as long-term steroid use can influence muscle bulk, bone strength, glucose tolerance and immune function.

The combination of immunotherapy and chemotherapy is now integrated as a standard of care in the first-line treatment setting of NSCLC.^103^ Although such combinations result in improved response rates, PFS and OS (regardless of PD-L1 status) compared with chemotherapy only, the toxicity rates are higher. Although sequential chemotherapy and immunotherapy might therefore seem a better option for older patients to reduce toxicity, appropriately selected older patients might benefit in some cases from combination strategies. It is therefore imperative to adequately assess older patients within this treatment scenario for a frailty or prefrailty status in order to avoid over- or under-treatment and to determine which patients will be able to tolerate the combination. Older patients are more prone to experience chemotherapy toxicity and are more likely to discontinue chemotherapy as a result;^104^ determining the aetiology of a given toxicity and managing it appropriately can be even more challenging when combining chemotherapy and immunotherapy. The major concern is that toxicities cause a functional decline that results in a loss of independence and a poorer QoL.

In conclusion, there is an essential need to generate data to address the use of immunotherapy in the older population as a whole, including in vulnerable and pre-frail patients.^105^ These data should include functional measures of frailty such as the G8, with a formal CGA in patients identified as vulnerable. Endpoints should not only be based around survival or response to treatment shown by imaging but should also include patient-reported outcomes such as maintaining QoL, which might be a more relevant goal in older patients. In addition, a further consideration is the potential impact of immunosenescence on immunotherapy. To this end, various biological markers and tests for immunosenescence could be incorporated into clinical trials to help determine whether the changes in the immune system associated with ageing have any impact on treatment efficacy and/or toxicity. Such assays include an assessment of T-cell phenotype, including the presence of circulating T_reg_ cells and CD8^+^/CD28^–^ T-cells and response to antigen challenge using EliSpot; the presence of autoantibodies; the presence of inflammatory markers, including the neutrophil to lymphocyte ratio and levels of CRP and IL-6; and an assessment of the stool microbiome for Firmicutes and Bacteroides species. Conducting such trials, however, can be difficult due to the heterogeneity of this population and the complex clinical variables. In addition, pharmaceutical companies might be less interested in focussing their studies on older patients or those with comorbidities where higher rates of adverse events are often encountered. In this regard, a good methodological compromise might be to design Phase 2 studies focusing on such patient populations, or to include specific preplanned subgroup analysis on older patients in pivotal randomised trials. Additionally, functional endpoints and patient-reported outcomes for older individuals could be included as exploratory or secondary endpoints in registration trials. Lastly, real-world data are an invaluable, readily available resource and should be collected and shared to help inform decision making when discussing treatment in these patient groups.

## Data Availability

Not applicable.
